# Comparison of post-activation performance enhancement in the lower limbs of short sprinters based on two velocity loss thresholds

**DOI:** 10.3389/fspor.2026.1772269

**Published:** 2026-03-11

**Authors:** Xiaohan Wang, Sidan Lu, Xinsong Cheng, Junjie He, Jianheng Wei, Qihao Sun, Tian Zhou, Yin Yu

**Affiliations:** 1 School of Sports Training, Wuhan Sports University, Wuhan, China; 2 Research Center for High-Quality Development of Characteristic Competitive Sports, Wuhan Sports University, Wuhan, China

**Keywords:** 30 m sprint, countermovementjump, post-activation potentiation enhancement, recovery time, velocity loss

## Abstract

**Objective:**

The present study examined the effects of post-activation performance enhancement (PAPE) induced by different velocity loss (VL) thresholds on lower-limb explosive performance in sprinters under velocity-based resistance training (VBT). A secondary aim was to identify the optimal VL thresholds (5% and 15%) and recovery time points (PRE, 4, 8, 12, and 16 min) for maximizing PAPE responses.

**Methods:**

Using a randomized crossover design, fifteen male sprinters completed two PAPE protocols consisting of deep squats performed at 85% 1RM with two VL thresholds (5% and 15%). Countermovement jump (CMJ) performance (jump height, relative power, and vertical impulse) and 30 m sprint performance (total time used and average speed) were assessed at each time point. The total number of squat repetitions completed under each VL condition was also recorded.

**Results:**

In the 5% VL condition, significant improvements were observed in CMJ jump height (*P* = 0.01), relative power (*P* = 0.009), vertical impulse (*P* = 0.016) at 8 min post-intervention. In addition, both total sprint time and mean speed showed significant changes at 4 min (*P* = 0.014; *P* = 0.030) and 8 min (*P* = 0.011; *P* = 0.006). In contrast, no significant changes in CMJ variables were found at any time point in the 15% VL condition. However, total sprint time and mean speed were significantly improved at 8 min post-intervention (*P* = 0.002; *P* = 0.004). The total number of squat repetitions was significantly lower in the 5% VL condition compared with the 15% VL condition (*P* = 0.003 vs. *P* = 0.042).

**Conclusion:**

Under two sets of deep squats at 85% 1RM, 5% VL was associated with CMJ improvement at 8 min and 30 m sprint improvement at 4 and 8 min with fewer repetitions, whereas 15% VL improved sprint performance mainly at 8 min with no clear CMJ enhancement.

## 1 Introduction

Post-activation potentiation (PAP) is a short-term increase in neuromuscular function after a high-intensity conditioning activity (CA). It is linked to neurophysiological and contractile mechanisms, such as higher motor neuron excitability and phosphorylation of myosin regulatory light chains. When these mechanisms lead to a measurable improvement in voluntary explosive performance, the outcome is referred to as post-activation performance enhancement (PAPE) (1–3). Myosin regulatory light chain phosphorylation can increase Ca^2^⁺ sensitivity of the contractile apparatus, thereby enhancing force output and rate of force development. Heightened neural drive may also support faster voluntary force production (2–4).

Lower limb strength is important for sprint performance. During acceleration, sprinters must apply high horizontal ground-reaction forces to increase speed. At maximal velocity, they must produce large support forces that are mainly vertical to maintain step-to-step speed (5). PAPE based warm-up strategies have been reported to acutely improve explosive lower limb performance in sprinters, which may influence sprint outcomes (6, 7).

In previous studies, PAPE is often induced using fixed-repetition schemes or percentage-based training (PBT), both of which are widely used to prescribe resistance loads (8, 9). However, fixed-repetition protocols do not account for day-to-day fluctuations in athlete readiness, and percentage-based loads (%1RM) may not reflect current capacity on a given day. As a result, these approaches can lead to inconsistent fatigue and potentiation responses, making it difficult to optimize the potentiation-fatigue balance required for acute PAPE in sprinters (8–12).

To address these limitations, velocity-based training (VBT) has been proposed as a more precise alternative. VBT uses real-time velocity feedback to monitor and regulate load intensity, repetition number, and neuromuscular fatigue (13, 14). Compared with traditional PBT, VBT allows direct quantification of fatigue and potentiation responses during the CA (10, 15). Movement velocity is closely linked to relative load and fatigue accumulation; thus, tracking velocity in real time enables immediate adjustments to better manage the potentiation-fatigue balance (16, 17).

A key feature of VBT is the use of velocity loss (VL) thresholds, the percentage decline in velocity from the fastest repetition within a set, as a reliable marker of accumulated neuromuscular fatigue (18, 19). Previous studies have used VL thresholds to standardize heavy-set volume and control fatigue during squat-based CAs (19–21). Incorporating VL thresholds allows precise control of training intensity and volume, which may reduce excessive fatigue while producing acute performance enhancements comparable to traditional methods (16, 22, 23).

Evidence supporting VL-controlled CAs for acute PAPE remains limited, particularly in sprint-trained athletes. Most available studies have used squat based CAs (e.g., 85%1RM) with low to moderate VL thresholds and have assessed performance at multiple recovery time points to describe the time course of the response (7). Evidence supporting mainly from a small number of athlete groups, including track and field, team sports (e.g., volleyball), and combat sports (e.g., boxing), and the outcomes have focused largely on jumping or general measures of explosive performance (7, 24–26). Within this context, we used VL thresholds as the main variable to define the CA. This allowed us to track the decline in movement velocity during the set in real time and compare acute PAPE responses across VL conditions.

Accordingly, VL thresholds should be selected to regulate fatigue accumulation during the CA and to support a potentiation–fatigue balance that favors net PAPE. VL is a validated marker of neuromuscular fatigue, and larger VL is typically associated with greater perceptual, metabolic, and neuromuscular fatigue responses (19, 27, 28). Therefore, we used 5% VL as a low-fatigue condition and 15% VL as a moderate-fatigue condition. This contrast is practically relevant for sprinting because sprint performance can be easily masked by residual fatigue. Both thresholds also fall within the low-to-moderate VL range that has been directly tested in PAPE studies using heavy squats, including designs that examined 5%–15% VL conditions (7, 24).

Evidence is still limited on how VBT can optimize acute PAPE in sprint-trained athletes. It is also unclear how different VL thresholds during a squat-based CA affect subsequent sprint and jump performance (7, 29, 30). The aim of this study was to compare the acute effects of two VL thresholds (5% and 15%) on PAPE responses in trained sprinters. We hypothesized that the 5% VL condition would produce greater acute improvements in sprint and jump performance than the 15% VL condition.

## 2 Materials and methods

### 2.1 Subjects

The required sample size was estimated *a priori* using G*Power 3.1 (31). The calculation was based on a repeated-measures crossover design in which each participant completed both VL conditions and served as his own control. Accordingly, a two-way repeated-measures ANOVA (condition  ×  time) was assumed, with a moderate effect size (f = 0.25), an alpha level of 0.05, and statistical power (1−*β*) of 0.80. The analysis indicated that a minimum of 12 participants was required. Ultimately, 15 male sprinters from the Hubei (China) track and field team were recruited, which was considered sufficient to ensure adequate statistical power.

Participants met the following inclusion criteria: (1) National Level II athletes or higher in sprint events (100 m, 200 m, or 400 m); (2) at least two years of systematic sprint training experience and prior experience with weighted deep squat training; and (3) no history of sports-related injuries or illnesses within the previous six months, and no neurological or psychiatric disorders that could influence experimental outcomes. The basic characteristics of the participants are presented in Table 1.

**Table 1:** Basic information about the subjects (*N* = 15).

Age (y)	Height (cm)	Weight (kg)	Body Fat (%)	Training Years	Squat 1RM (kg)
18.33 ± 1.54	179.13 ± 3.16	67.99 ± 7.54	10.55 ± 2.06	4.20 ± 1.42	126.33 ± 21.50

All participants provided informed consent prior to participation. The study was conducted in accordance with the Declaration of Helsinki and was approved by the Research Ethics Committee of Wuhan Sports University (approval number: 2025115). Electronic informed consent was obtained via an online system, and written informed consent was reconfirmed and collected in person before the experiment. The ethical approval became effective on November 1, 2025, and all experimental procedures strictly complied with the requirements of the institutional review board.

### 2.2 Experimental design

This study adopted a randomized, repeated-measures crossover design to examine the effects of deep squat induced PAPE on lower-limb explosive performance in male sprinters under two VL threshold conditions (5% and 15%). All participants first completed a 1RM back squat test and baseline assessments to determine individual loading parameters. They subsequently performed two PAPE testing sessions, each corresponding to one VL threshold condition.

The two PAPE sessions were completed in different testing weeks, with at least 7 days between sessions to minimize residual fatigue and carryover effects. After each session, Countermovement jump (CMJ) and 30 m sprint performance were assessed on separate testing days.

To control for potential circadian rhythm effects, all testing sessions were scheduled at the same time of day. Acute potentiation effects were quantified by comparing CMJ performance before and after each PAPE intervention. This experimental design allowed for a precise comparison of PAPE responses between different VL threshold conditions and provided a framework for examining the interaction between load-induced fatigue and potentiation effects.

### 2.3 Procedures

#### 2.3.1 Warm-up and test methods

##### 2.3.1.1 Warm-up

Participants completed a standardized warm-up protocol prior to testing. This consisted of 5 min of myofascial release, followed by 5 min of light jogging, and 5 min of dynamic stretching. The dynamic stretching sequence included: (1) hip flexion with straight-leg drop, marching Superman stretch, marching knee-to-heel lifts, hand-to-foot crawling, and knee-to-heel lifts, with each exercise performed over 10 m for two sets; (2) 90/90 hip stretching combined with kneeling hip thrusts (two sets of eight repetitions); and (3) maximal stretching performed over a distance of 15 m for one set.

##### 2.3.1.2 1RM test

Equipment and Measurement: Deep squat 1RM was assessed using a GymAware linear velocity sensor (Kinetic Performance Technologies, Canberra, Australia) to record barbell velocity (MPV) during incremental loading.
Testing Procedure: Squat 1RM was directly determined using an incremental loading protocol. Following a standardized warm-up, participants performed six repetitions at 40 kg, with subsequent load increments guided by MPV. Resistance was increased by 5–10 kg per set until MPV dropped below 0.70 m·s⁻^1^, after which increments were refined to 1–5 kg to accurately identify the 1RM while minimizing cumulative fatigue (23). A 3–5 min passive rest was provided between sets (32). The 1RM was defined as the heaviest load successfully lifted for a single repetition with proper technique, as standardized by the NSCA guidelines (33). Two experienced spotters monitored all trials to ensure safety and technical proficiency.

##### 2.3.1.3 CMJ test

Equipment and Measurement: CMJ jump height, relative power, and vertical impulse were assessed using a Kistler 3D force platform (Kistler Instrumente AG, Switzerland) operating at a sampling frequency of 1,000 Hz. Test–retest reliability across weeks, based on the pre-exercise baseline values from the two testing weeks, was acceptable to excellent (ICC = 0.47–0.96; CV = 1.8–5.6%).
Testing Procedure: During testing, participants stood on the force platform with their hands placed on the waist and feet positioned approximately shoulder-width apart. Upon receiving the verbal command, participants performed a rapid countermovement by descending into a squat position, with the thighs approaching or reaching a position parallel to the ground, followed immediately by a maximal vertical jump. Throughout the movement, participants were instructed to maintain an upright trunk posture, keep the chest lifted, and engage the core musculature. During the landing phase, both feet were required to contact the platform simultaneously, with the knees flexed naturally (approximately 90–150°) to attenuate impact forces. Participants were instructed to stabilize their posture and maintain balance after landing.

##### 2.3.1.4 30 m sprint test

Equipment and Measuremen: The 30 m sprint test was performed using a motorized sprint resistance device (1080 Sprint, Ningbo, China). Sprint time and mean running velocity over 30 m were recorded for analysis.
Testing Procedure: Before each trial, the device was set to a constant minimal resistance (1 kg) to maintain cable tension. After a standardized warm-up, participants completed a maximal 30 m sprint from a three-point stance. To eliminate cable slack and improve measurement reliability, the device applied a constant minimal baseline tension of 1 kg (34). Participants were instructed to maintain their habitual sprint technique. Relative to the group's mean body mass, this setting corresponded to approximately 1.47% body mass, well within commonly defined light-load conditions (<10% body mass) intended to preserve sprint-specific kinematics (35). Accordingly, the procedure is described as a standardized motorized sprint test under minimal baseline tension, likely approximating near-unresisted sprinting capacity.

##### 2.3.1.5 Experimental overview

All tests and interventions were conducted at the Hubei Provincial Research Center for High-Quality Development of Characteristic Competitive Sports (China).The 1RM assessment, baseline CMJ, and 30 m sprint tests were performed on separate days, with at least 72 h between tests.

On the baseline testing day, following a standardized 15 min warm-up, participants rested for 5 min and then performed three CMJs with 10 s of rest between trials. The 30 m sprint test was conducted on a separate day and consisted of two maximal 30 m sprints separated by 5 min of passive recovery. Jump height, relative power, and vertical impulse were recorded for CMJ, while total sprint time and average velocity were recorded for the 30 m sprint. For each variable, the best performance was retained as the baseline value. This selection rule was applied consistently at all subsequent time points. Participants were instructed to avoid high-intensity training on the day preceding each test and to refrain from consuming stimulants such as caffeine or alcohol that could influence performance.

On the experimental testing days, participants completed the same standardized warm-up, followed by a 3 min rest period. The PAPE intervention consisted of two sets of deep squats performed at 85% 1RM, with a 1 min rest interval between sets. During each squat repetition, a tester secured an elastic band at the initial squat position to ensure a consistent depth, with the thighs reaching a position parallel to the ground. A GymAware linear velocity sensor was positioned on the floor to the right side of the squat rack, with the tether aligned perpendicular to the ground, to record MPV in real time.

Participants were verbally encouraged to perform each concentric phase as explosively as possible. Mean propulsive velocity for each repetition was simultaneously displayed in the GymAware software and provided to the athlete via auditory feedback. VL was calculated using the equation: stopping velocity = initial velocity×(1−VL) (7, 18, 36). Each set was terminated when the repetition velocity fell below the target threshold, indicating that the prescribed VL had been exceeded.

**Figure 1:**
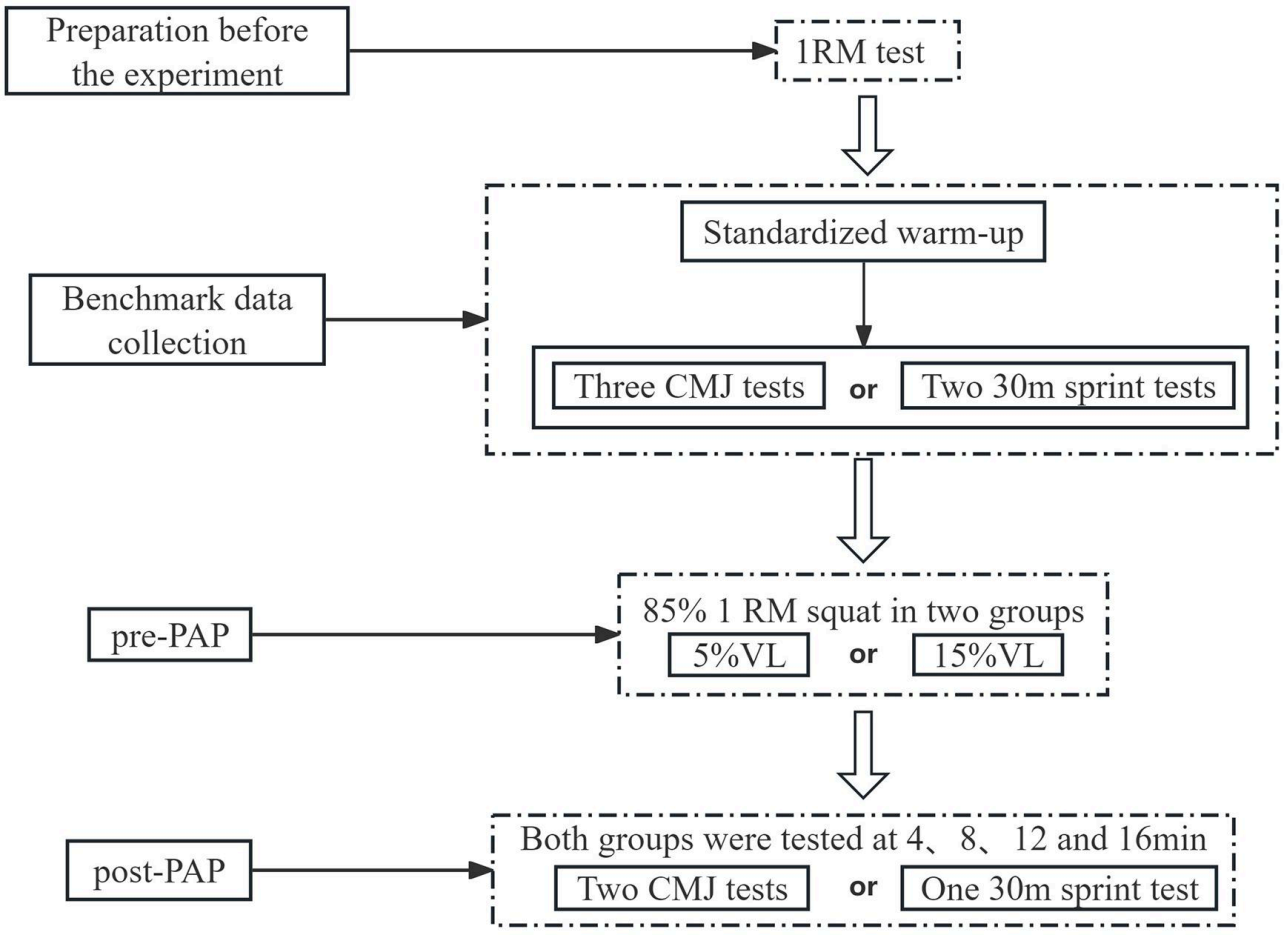
Experimental procedure. CMJ, Countermovement jump, min, minutes.

After completing the two sets of deep squats, subjects will perform CMJ or 30 m sprint tests within the post-recovery PAPE time window (38). At each time point, CMJ was performed twice, and the best value was retained, whereas the 30 m sprint was performed once. All performance variables were collected using the same procedures as in the baseline testing (Figure 1).

## 3 Statistical analysis

The experimental data were summarized by Excel and the effects of the two VL thresholds and recovery time on the experimental data were evaluated using SPSS 26.0 software (two-factor repeated measures ANOVA). The experimental results were processed 2 × 5 (VL × recovery time) using repeated measures two-way ANOVA. The significance level *α* was set at 0.05 during the test of variance throughout the experimental data for significance (*P* < 0.05); highly significant (*P* < 0.01).

Mauchly's test of sphericity was performed for CMJ jump height, relative power, vertical impulse, and total sprint time and average velocity during the 30 m sprint across different VL thresholds and recovery time points. When the sphericity assumption was met (*P* > 0.05), unadjusted two-way ANOVA results were reported. When the assumption was violated (*P* ≤ 0.05), degrees of freedom were corrected using the Greenhouse–Geisser adjustment. One-way ANOVA was used to compare the number of resisted deep squat repetitions performed under different VL conditions. Differences in CMJ performance between baseline and post-intervention time points, as well as differences in squat repetition number between the two VL thresholds, were analyzed using paired-samples t-tests. Effect sizes were calculated using Cohen's d and interpreted as follows: <0.2, trivial; 0.2–0.6, small; 0.6–1.2, moderate; and >1.2, large (39).

## 4 Results

### 4.1 CMJ performance

**Figure 2:**
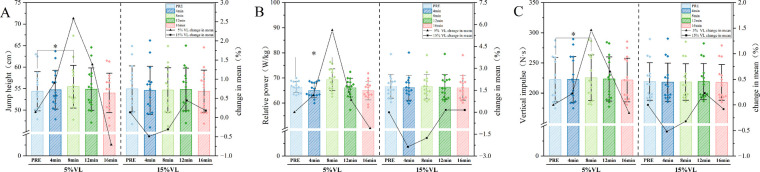
CMJ performance metrics across time points under two VL thresholds. (**A**: Jump height; **B**: Relative power; **C**:Vertical impulse). Data are presented as mean ± SD. * indicates significant difference from PRE at *p* ≤ 0.05.

Data at each time point (PRE, 4, 8, 12, and 16 min) under different VL thresholds were analyzed using a two-way repeated-measures ANOVA (VL × time). Mauchly's test of sphericity indicated a violation of the sphericity assumption (*p* < 0.05); therefore, corrected results were reported. The main effect of VL threshold was not significant for jump height (F = 0.001, *p* = 0.971), relative power (F = 0.002, *p* = 0.964), or vertical impulse (F = 0.184, *p* = 0.672). A significant main effect of time was observed for relative power (*p* < 0.001) and vertical impulse (*p* = 0.046), whereas jump height did not show a significant time effect after correction (F = 3.948, *p* = 0.18). A significant VL × time interaction was found only for relative power (F = 3.779, *p* = 0.033). No significant interaction effects were observed for jump height (F = 2.758, *p* = 0.62) or vertical impulse (F = 2.131, *p* = 0.116). *post hoc* analyses revealed that, in the 5% VL condition, all three CMJ variables showed significant increases at 8 min post-intervention (jump height: *p* = 0.010; relative power: *p* = 0.009; vertical impulse: *p* = 0.016). In contrast, no significant changes were observed at any time point in the 15% VL condition (Tables 2, 3; Figure 2).

**Table 2:** CMJ and 30 m sprint test results before and after the two VL-inducing exercise interventions at each time point (*N* = 15).

Test items		Statistical indicators	5% VL group					15% VL group				
			PRE	4min	8min	12min	16min	PRE	4min	8min	12min	16min
CMJ	Jump Height	T	/	1.735	3.058	1.646	−1.081	/	−1.49	−1.363	1.055	0.21
		P	/	0.108	0.01*	0.126	0.301	/	0.162	0.198	0.312	0.837
		M（cm）	54.40	54.76	55.56	54.99	54.00	54.89	54.59	54.67	55.03	54.91
		SD（cm）	4.61	4.54	4.93	5.00	4.66	5.39	5.64	5.16	5.25	5.45
	Relative power	T	/	1.233	3.139	0.721	−0.876	/	−2.118	−1.215	0.108	0.09
		P	/	0.241	0.009*	0.485	0.398	/	0.056	0.248	0.916	0.93
		M（W/kg）	66.02	66.79	69.72	66.57	65.29	66.12	64.56	64.96	66.22	66.21
		SD（W/kg）	2.52	3.03	4.24	3.92	3.72	2.46	2.96	3.14	4.23	4.50
	Vertical impulse	T	/	0.996	2.801	1.548	−0.327	/	−1.905	−1.538	0.463	−0.243
		P	/	0.339	0.016*	0.148	0.749	/	0.081	0.15	0.651	0.812
		M（N·s）	222.28	222.77	225.52	223.74	221.92	223.95	222.79	223.23	224.46	223.77
		SD（N·s）	36.84	37.94	38.09	36.65	36.00	37.95	38.34	37.27	36.07	38.51
30 m sprint	Total time used	T	/	−2.813	0.682	−1.878	0.798	/	1.852	−2.635	−1.482	−1.665
		P	/	0.014*	0.011*	0.081	0.438	/	0.085	0.02*	0.161	0.118
		M（s）	3.050	2.988	3.002	3.022	3.060	3.055	3.086	3.022	3.019	3.021
		SD（s）	0.061	0.117	0.088	0.047	0.077	0.065	0.067	0.093	0.099	0.096
	Average speed	T	/	2.414	3.198	2.033	1.821	/	−1.655	2.264	1.008	1.347
		P	/	0.03*	0.006*	0.062	0.09	/	0.12	0.04*	0.331	0.2
		M（m/s）	7.794	7.869	7.892	7.855	7.861	7.858	7.788	7.941	7.906	7.927
		SD（m/s）	0.211	0.222	0.222	0.217	0.228	0.187	0.169	0.201	0.214	0.240

**Table 3:** Effect sizes (Cohen's d, 95% CI) for changes from PRE in CMJ and 30 m sprint outcomes under 5% and 15% VL.

Test items		VL(%)	Effect size with 95%CI			
			4min	8min	12min	16min
CMJ	Jump Height	5	0.448 (−0.076, 0.972)	0.790 (0.150, 1.430)	0.425 (−0.093, 0.943)	−0.279(−0.778, 0.220)
		15	−0.385(−0.899, 0.129)	−0.352(−0.860, 0.156)	0.272(−0.231, 0.775)	0.054 (−0.435, 0.543)
	Relative power	5	0.318(−0.198, 0.834)	0.810 (0.169, 1.451)	0.186(−0.320, 0.692)	−0.226(−0.720, 0.268)
		15	−0.547(−1.096, 0.002)	−0.314(−0.826, 0.198)	0.028(−0.460, 0.516)	0.023(−0.463, 0.509)
	Vertical impulse	5	0.257(−0.245, 0.759)	0.723 (0.108, 1.338)	0.400(−0.115, 0.915)	−0.084(−0.573, 0.405)
		15	−0.492(−1.002, 0.018)	−0.397(−0.915, 0.121)	0.120(−0.395, 0.635)	−0.063(−0.550, 0.424)
30 m sprint	Total time used	5	−0.726(−1.341, −0.111)	0.176(−0.334, 0.686)	−0.485(−0.993, 0.023)	0.206(−0.301, 0.713)
		15	0.478(−0.038, 0.994)	−0.680(−1.272, −0.088)	−0.383(−0.897, 0.131)	−0.430(−0.947, 0.087)
	Average speed	5	0.623 (0.034, 1.212)	0.826 (0.184, 1.468)	0.525(−0.009, 1.059)	0.470(−0.046, 0.986)
		15	−0.427(−0.943, 0.089)	0.584 (0.068, 1.100)	0.26(−0.242, 0.762)	0.348(−0.158, 0.854)

### 4.2 30 m sprint performance

**Figure 3:**
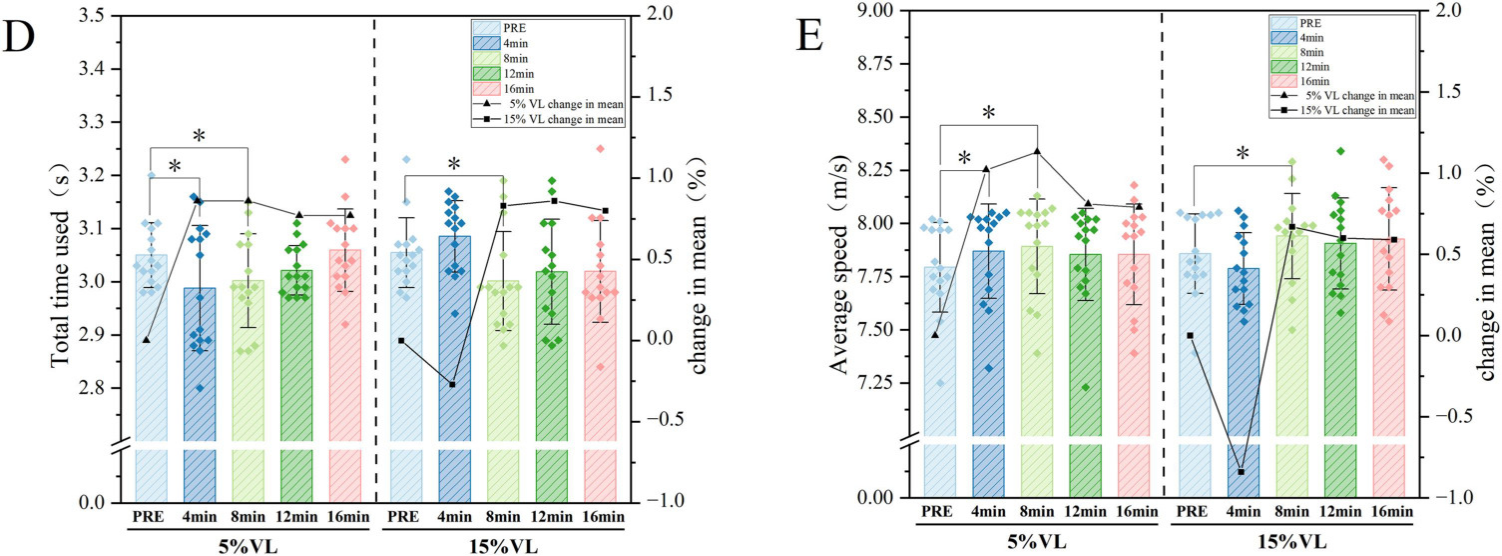
Sprint performance following VL-controlled PAPE protocols. (**D**: Total time used; **E**: Average speed). Data are presented as mean ± SD. * indicates significant difference from PRE at *p* ≤ 0.05.

Data at each time point (PRE, 4, 8, 12, and 16 min) under different VLthresholds were analyzed using a two-way repeated-measures ANOVA (VL × time). Mauchly's test of sphericity indicated a violation of the sphericity assumption (*p* < 0.05); therefore, corrected results were reported. The main effect of VL threshold was not significant for total sprint time (F = 0.291, *p* = 0.594) or average speed (F = 0.195, *p* = 0.663). A significant main effect of time was observed for total sprint time (F = 2.936, *p* = 0.037) and average speed (F = 3.696, *p* = 0.007). A significant VL × time interaction was found for total sprint time (F = 4.982, *p* = 0.003), whereas no significant interaction effect was observed for average speed (F = 2.242, *p* = 0.67). *post hoc* analyses showed that, under the 5% VL condition, total sprint time used and average speed were significantly altered at both 4 min (*p* = 0.014; *p* = 0.030) and 8 min (*p* = 0.011; *p* = 0.006) post-intervention. In contrast, under the 15% VL condition, significant changes in total sprint time and average speed were observed only at 8 min post-intervention (*p* = 0.02 and *p* = 0.04, respectively) (Tables 2, 3; Figure 3).

### 4.3 Results of deep squat counts

The analysis of the number of deep squat repetitions in Group 1 showed a significant difference between the 5% VL and 15% VL conditions. Under both the CMJ and 30 m sprint protocols, the number of deep squats performed at 5% VL was significantly lower than that at 15% VL (*p* < 0.001). No significant difference was observed between the CMJ−5% VL and CMJ-15% VL conditions (*p* > 0.05). Similar results were observed in Group 2. No significant difference was found between the CMJ-5% VL and CMJ-15% VL conditions (*p* > 0.05). However, during the 30 m sprint protocol, the number of deep squats performed at 5% VL was significantly lower than that at 15% VL (*p* < 0.001). When the total number of deep squat repetitions was pooled across both groups, the overall number of repetitions performed under the 5% VL condition was significantly lower than that under the 15% VL condition (*p* < 0.001) (Table 4).

**Table 4:** Statistics of total deep squats.

Test items	VL(%)	Squats (reps)		
		Group 1	Group 2	Sum of the two groups
CMJ	5	3.00 ± 0.71b	3.15 ± 0.99b	6.15 ± 1.28b
	15	4.85 ± 0.99a	4.77 ± 1.01a	9.62 ± 1.50a
30 m sprint	5	2.77 ± 0.73b	3.15 ± 0.90b	5.92 ± 1.38b
	15	4.69 ± 1.38a	4.38 ± 1.12a	9.08 ± 2.06a

## 5 Discussion

This study examined whether a 5% or 15% VL threshold during 85% 1RM deep squats better induces PAPE. Direct comparisons are limited. Overall, 5% VL produced greater high-speed performance gains (CMJ at 8 min; 30 m sprint at 4 and 8 min), whereas 15% VL improved only the 30 m sprint at 8 min, indicating VL and time dependent PAPE responses.

Previous studies have suggested that athletes’ 1RM and fatigue status fluctuate over time, which may reduce the accuracy of fixed load prescriptions based on PBT and potentially compromise PAPE induction (10). In this context, VBT has been proposed as an alternative approach that may help individualize load prescription by using real time barbell velocity feedback to inform load–velocity profiling (13, 14). Encouraging maximal voluntary intent during the concentric phase, together with velocity feedback, may improve movement consistency and the accuracy of day to day load prescription. VBT may be relevant for stretch–shortening cycle (SSC) based tasks such as jumping and sprinting because it encourages high effort intent and helps maintain the intended movement velocity profile during the conditioning set (20, 37–39). Real time velocity feedback can support this intent and provides an objective signal for stopping sets before velocity drops excessively, rather than relying on a fixed repetition target (20, 40). VL during resistance exercise is closely related to acute fatigue responses, so limiting the decline in velocity may reduce the likelihood that early fatigue masks potentiation (19). Evidence from VLcontrolled protocols shows that larger velocity losses are typically accompanied by greater perceived effort and metabolic stress, which supports VLas a practical way to regulate the conditioning dose (32, 41).

Studies on VBT have shown a consistent relationship between %1RM and movement velocity across various loading ranges (10, 13). This relationship forms the basis of the VL threshold, a widely used method in resistance training to regulate set termination and monitor fatigue (17, 18). Compared to fixed repetition prescriptions, VL based set termination allows for adjustments based on real time velocity decline, which better accounts for day to day and individual variability (42). Importantly, using fewer repetitions is not the same as controlling fatigue exposure. A fixed repetition target can reduce volume, but repetition capacity at the same relative load can vary across athletes and across days, so fatigue exposure may still differ (19, 38). VL addresses this by ending the set when performance has declined by a similar relative amount, which is commonly treated as a practical marker of within set neuromuscular fatigue and proximity to failure (18). This feature makes VL useful in acute PAPE protocols, where the goal is to manage fatigue rather than accumulate training volume (43).

It is important to note that performance changes following CA should not be attributed to fatigue alone, as factors like changes in motor coordination or jumping strategy also affect outcomes like CMJ performance (44). Studies show that higher VL thresholds often lead to greater fatigue responses (45). For example, Weakley et al. (41) found that higher VL was associated with increased ratings of perceived exertion and blood lactate, as well as decreases in movement velocity and jump height. Similarly, Sánchez-Medina et al. (19) reported strong correlations between VL and neuromuscular fatigue indicators (r = 0.91– 0.97). Although we did not measure physiological fatigue markers, the higher VL condition was linked to greater short term decrements in CMJ and sprint performance, reflecting a higher fatigue cost. Consistent with this, the 15% VL condition required more deep squat repetitions than the 5% VL condition, which likely increased early fatigue exposure. Excessive fatigue may limit rapid force production, thus reducing improvements in explosive performance (18), which might explain why the 15% VL condition showed a delayed or smaller net PAPE effect compared to the 5% VL condition.

Beyond fatigue, the two VL thresholds also differed in how much speed dropped during the conditioning squat. A smaller VLlikely helped keep the movement quality more stable and limited early fatigue (26). This may have made it easier for net PAPE to appear in CMJ and sprint tests within the early recovery window (46).This could help explain the more favorable acute PAPE pattern observed under the 5% VL condition (39). Yuan et al. (7) also found that during deep squat training with different VL thresholds, only the low VL condition (5%) resulted in significant improvements in CMJ height (ES = 0.73), peak power output (ES = 0.73), and impulse (ES = 0.72). Similarly, Tsoukos et al. (47) reported that in a velocity-based repetition control scheme during heavy bench press (80% 1RM), terminating sets at a lower VL (10%) led to earlier and more sustained performance improvements than a higher VL (30%), with fewer repetitions and lower volume load. Together, these findings suggest that limiting VLmay reduce fatigue accumulation and preserve a task-relevant stimulus, facilitating earlier net PAPE expression.

However, evidence is not entirely uniform. In a squat based PAPE protocol also performed at 85% 1RM, Li et al. (26) reported that a moderate VL target (20%) elicited an earlier improvement in 0–20 m sprint performance at 4 min, whereas the smaller VL target (10%) showed its clearest improvement at a later time point (16 min). A plausible explanation is that the apparent “optimal” VL is highly sensitive to the performance task and the recovery window sampled (26). Short sprint acceleration (0–20 m) may benefit from a slightly greater conditioning dose to transiently increase early-phase force production, even if this comes with a higher fatigue cost (6, 48). In contrast, our outcomes (CMJ and 30 m sprint) and our VL comparison (5% vs. 15%) likely placed greater emphasis on preserving movement velocity and limiting fatigue to allow net PAPE to emerge within the 4–8 min window. Accordingly, different VL thresholds can appear “optimal” when the conditioning dose required to elicit potentiation, the fatigue tolerance of the outcome task, and the timing of post-activation testing differ across studies (29, 49, 50).

PAPE follows a time dependent pattern because potentiation and fatigue coexist after the CA and their balance shifts during recovery. These two processes coexist and exert opposing effects on force production (4, 6). under the 15% VL condition. Several meta-analyses have investigated the optimal recovery interval for PAPE (6, 51). Very short recovery (0–3 min) is often linked to impaired performance, whereas improvements are more consistently reported around 7–12 min (51–53). For jump outcomes, Kilduff et al. reported small but significant effects with rest intervals of 8–12 min (ES = 0.24) (51, 54), while Wilson et al. identified 7–10 min (ES = 0.70) as an optimal window (52). Our findings broadly align with this literature. In our protocol, the clearest improvements were observed at 8 min. Earlier responses were more dependent on the VL condition, which is consistent with greater variability in the early recovery phase when fatigue is more likely to influence performance (7, 29). Under 5% VL, sprint improved at 4 min, suggesting shorter recovery may suffice. Under 15% VL, improvements appeared later, indicating delayed net PAPE. Taken together, Differences in conditioning dose (load and volume), the nature of the CA, and the training status of participants can shift the balance between fatigue and potentiation and, therefore, the time course of net PAPE (6, 51, 52).

## 6 Limitations

The participants in this study were all male sprinters. Future studies are needed to confirm whether these findings also apply to female athletes. In addition, we did not collect physiological or neuromuscular markers during the CA or recovery; therefore, interpretations regarding fatigue, potentiation, and their balance are inferred from performance outcomes and VL control rather than directly verified.

## 7 Conclusion

This study compared PAPE responses between two VL thresholds (5% and 15%) during two sets of deep squats at 85% 1RM in trained male sprinters. Within the tested recovery windows, the 5% VL condition improved CMJ outcomes at 8 min and 30 m sprint performance at 4 and 8 min. It also required fewer squat repetitions than the 15% VL condition. The 15% VL condition improved sprint performance mainly at 8 min and did not enhance CMJ at any time point. These findings are specific to this cohort and protocol and should be interpreted within the tested loading and recovery windows. A lower VL threshold may help regulate conditioning volume when heavy squats are used for acute PAPE preparation in sprinters.

## Data Availability

The original contributions presented in the study are included in the article/Supplementary Material, further inquiries can be directed to the corresponding author.
