# Supplementary material for: Comparison of post-activation performance enhancement in the lower limbs of short sprinters based on two velocity loss thresholds
Source: Front Sports Act Living. 2026 Mar 11;8:1772269. doi: 10.3389/fspor.2026.1772269 (PMC13013364; doi:10.3389/fspor.2026.1772269)
Supplement: Supplementary file 1 [file Datasheet1.docx]

Supplementary Material

# 1 Supplementary Tables

**Table 1 Basic information about the subjects (N = 15)**

| Age (y) | Height (cm) | Weight (kg) | Body Fat (%) | Training Years | Squat 1RM (kg) |
| --- | --- | --- | --- | --- | --- |
| 18.33±1.54 | 179.13±3.16 | 67.99±7.54 | 10.55±2.06 | 4.20±1.42 | 126.33±21.50 |

1RM = one-repetition maximum (1RM).

**Table 2 CMJ and 30m sprint test results before and after the two VL-inducing exercise interventions at each time point (N=15)**

| **Test items** | | **Statistical**  **indicators** | **5% VL group** | | | | |  | **15% VL group** | | | | |
| --- | --- | --- | --- | --- | --- | --- | --- | --- | --- | --- | --- | --- | --- |
|  |  |  | **PRE** | **4min** | **8min** | **12min** | **16min** |  | **PRE** | **4min** | **8min** | **12min** | **16min** |
| CMJ | Jump Height | T | / | 1.735 | 3.058 | 1.646 | -1.081 |  | / | -1.49 | -1.363 | 1.055 | 0.21 |
|  |  | P | / | 0.108 | 0.01* | 0.126 | 0.301 |  | / | 0.162 | 0.198 | 0.312 | 0.837 |
|  |  | M（cm） | 54.40 | 54.76 | 55.56 | 54.99 | 54.00 |  | 54.89 | 54.59 | 54.67 | 55.03 | 54.91 |
|  |  | SD（cm） | 4.61 | 4.54 | 4.93 | 5.00 | 4.66 |  | 5.39 | 5.64 | 5.16 | 5.25 | 5.45 |
|  | Relative power | T | / | 1.233 | 3.139 | 0.721 | -0.876 |  | / | -2.118 | -1.215 | 0.108 | 0.09 |
|  |  | P | / | 0.241 | 0.009* | 0.485 | 0.398 |  | / | 0.056 | 0.248 | 0.916 | 0.93 |
|  |  | M（W/kg） | 66.02 | 66.79 | 69.72 | 66.57 | 65.29 |  | 66.12 | 64.56 | 64.96 | 66.22 | 66.21 |
|  |  | SD（W/kg） | 2.52 | 3.03 | 4.24 | 3.92 | 3.72 |  | 2.46 | 2.96 | 3.14 | 4.23 | 4.50 |
|  | Vertical impulse | T | / | 0.996 | 2.801 | 1.548 | -0.327 |  | / | -1.905 | -1.538 | 0.463 | -0.243 |
|  |  | P | / | 0.339 | 0.016* | 0.148 | 0.749 |  | / | 0.081 | 0.15 | 0.651 | 0.812 |
|  |  | M（N·s） | 222.28 | 222.77 | 225.52 | 223.74 | 221.92 |  | 223.95 | 222.79 | 223.23 | 224.46 | 223.77 |
|  |  | SD（N·s） | 36.84 | 37.94 | 38.09 | 36.65 | 36.00 |  | 37.95 | 38.34 | 37.27 | 36.07 | 38.51 |
| 30m sprint | Total time used | T | / | -2.813 | 0.682 | -1.878 | 0.798 |  | / | 1.852 | -2.635 | -1.482 | -1.665 |
|  |  | P | / | 0.014* | 0.011* | 0.081 | 0.438 |  | / | 0.085 | 0.02* | 0.161 | 0.118 |
|  |  | M（s） | 3.050 | 2.988 | 3.002 | 3.022 | 3.060 |  | 3.055 | 3.086 | 3.022 | 3.019 | 3.021 |
|  |  | SD（s） | 0.061 | 0.117 | 0.088 | 0.047 | 0.077 |  | 0.065 | 0.067 | 0.093 | 0.099 | 0.096 |
|  | Average speed | T | / | 2.414 | 3.198 | 2.033 | 1.821 |  | / | -1.655 | 2.264 | 1.008 | 1.347 |
|  |  | P | / | 0.03* | 0.006* | 0.062 | 0.09 |  | / | 0.12 | 0.04* | 0.331 | 0.2 |
|  |  | M（m/s） | 7.794 | 7.869 | 7.892 | 7.855 | 7.861 |  | 7.858 | 7.788 | 7.941 | 7.906 | 7.927 |
|  |  | SD（m/s） | 0.211 | 0.222 | 0.222 | 0.217 | 0.228 |  | 0.187 | 0.169 | 0.201 | 0.214 | 0.240 |

Note: * represents a significant difference at p≤0.05 compared to PRE (baseline value).

**Table 3 Effect sizes (Cohen’s d, 95% CI) for changes from PRE in CMJ and 30m sprint outcomes under 5% and 15% VL**

| **Test items** | | **VL(%)** | **Effect size with 95%CI** | | | |
| --- | --- | --- | --- | --- | --- | --- |
|  |  |  | 4min | 8min | 12min | 16min |
| CMJ | Jump Height | 5 | 0.448 (-0.076, 0.972) | 0.790 (0.150, 1.430) | 0.425 (-0.093, 0.943) | -0.279(-0.778, 0.220) |
|  |  | 15 | -0.385(-0.899, 0.129) | -0.352(-0.860, 0.156) | 0.272(-0.231, 0.775) | 0.054 (-0.435, 0.543) |
|  | Relative power | 5 | 0.318(-0.198, 0.834) | 0.810(0.169, 1.451) | 0.186(-0.320, 0.692) | -0.226(-0.720, 0.268) |
|  |  | 15 | -0.547(-1.096, 0.002) | -0.314(-0.826, 0.198) | 0.028(-0.460, 0.516) | 0.023(-0.463, 0.509) |
|  | Vertical impulse | 5 | 0.257(-0.245, 0.759) | 0.723(0.108, 1.338) | 0.400(-0.115, 0.915) | -0.084(-0.573, 0.405) |
|  |  | 15 | -0.492(-1.002, 0.018) | -0.397(-0.915, 0.121) | 0.120(-0.395, 0.635) | -0.063(-0.550, 0.424) |
| 30m sprint | Total time used | 5 | -0.726(-1.341, -0.111) | 0.176(-0.334, 0.686) | -0.485(-0.993, 0.023) | 0.206(-0.301, 0.713) |
|  |  | 15 | 0.478(-0.038, 0.994) | -0.680(-1.272, -0.088) | -0.383(-0.897, 0.131) | -0.430(-0.947, 0.087) |
|  | Average speed | 5 | 0.623(0.034, 1.212) | 0.826(0.184, 1.468) | 0.525(-0.009, 1.059) | 0.470(-0.046, 0.986) |
|  |  | 15 | -0.427(-0.943, 0.089) | 0.584(0.068, 1.100) | 0.26(-0.242, 0.762) | 0.348(-0.158, 0.854) |

**Table 4 Statistics of total deep squats**

| **Test items** | **VL(%)** | **Squats (reps)** | | | | |
| --- | --- | --- | --- | --- | --- | --- |
|  |  | **Group 1** |  | **Group 2** |  | **Sum of the two groups** |
| CMJ | 5 | 3.00 ± 0.71b |  | 3.15 ± 0.99b |  | 6.15± 1.28b |
|  | 15 | 4.85 ± 0.99a |  | 4.77 ± 1.01a |  | 9.62 ± 1.50a |
| 30m sprint | 5 | 2.77 ± 0.73b |  | 3.15 ± 0.90b |  | 5.92 ± 1.38b |
|  | 15 | 4.69 ± 1.38a |  | 4.38 ± 1.12a |  | 9.08 ± 2.06a |

Note:a indicates a significant difference in the number of deep squat repetitions compared to the 5% VL group; b indicates a significant difference in the number of deep squat repetitions compared to the 15% VL group.

## 2 Supplementary Figures


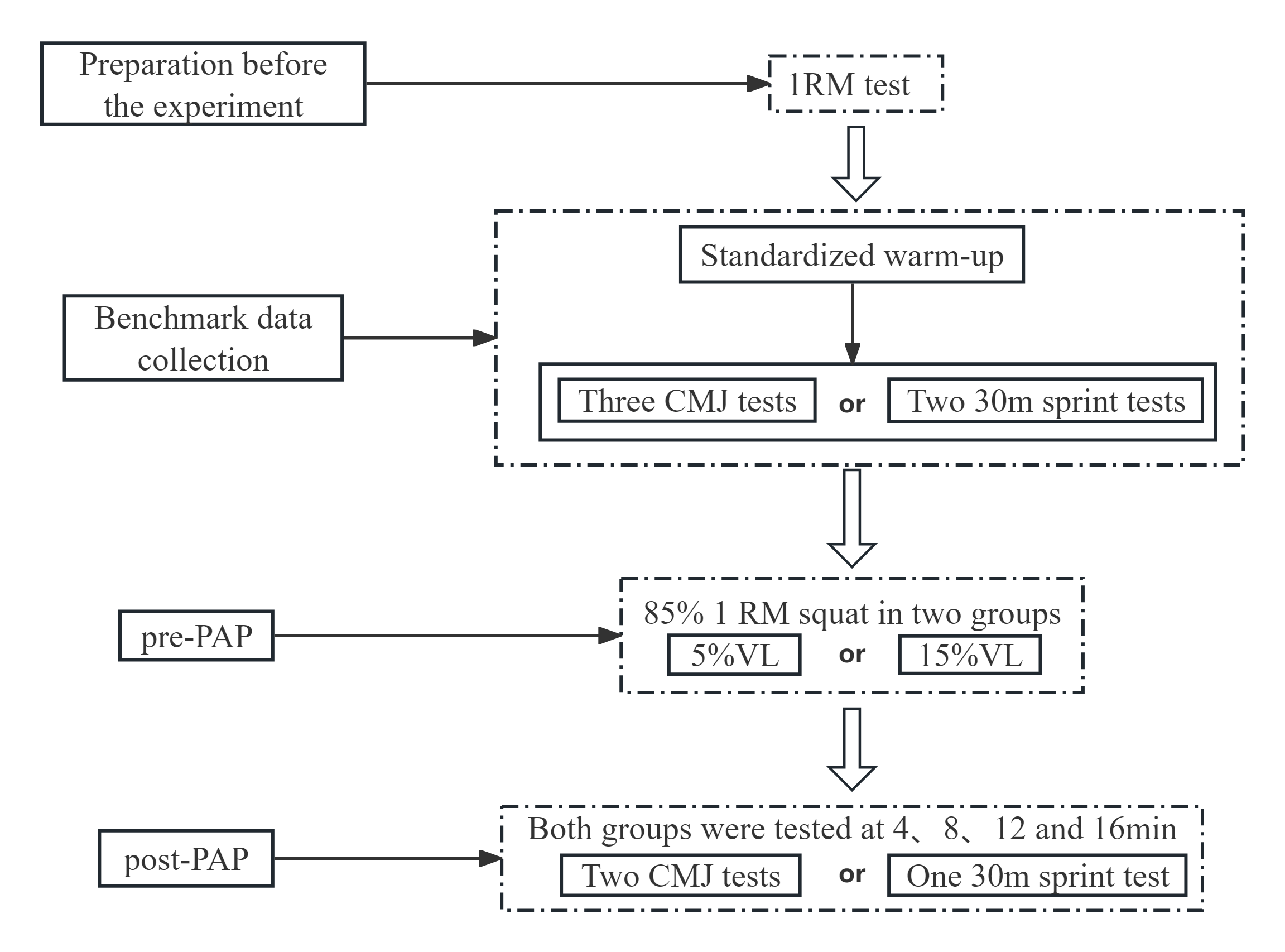


**Figure 1. Experimental procedure.** CMJ = Countermovement jump, min = minutes.


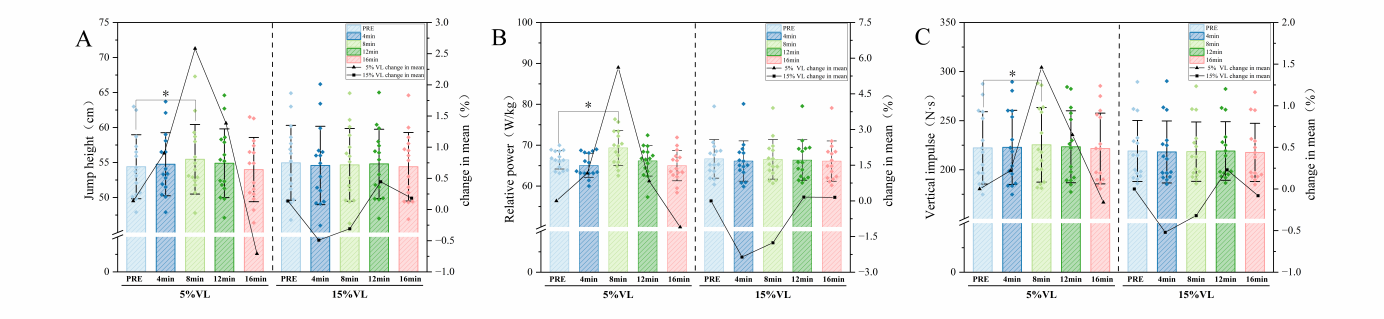
**Figure 2.** CMJ performance metrics across time points under two VL thresholds. (A: Jump height; B: Relative power; C :Vertical impulse) . Data are presented as mean ± SD. * indicates significant difference from PRE at p≤0.05.


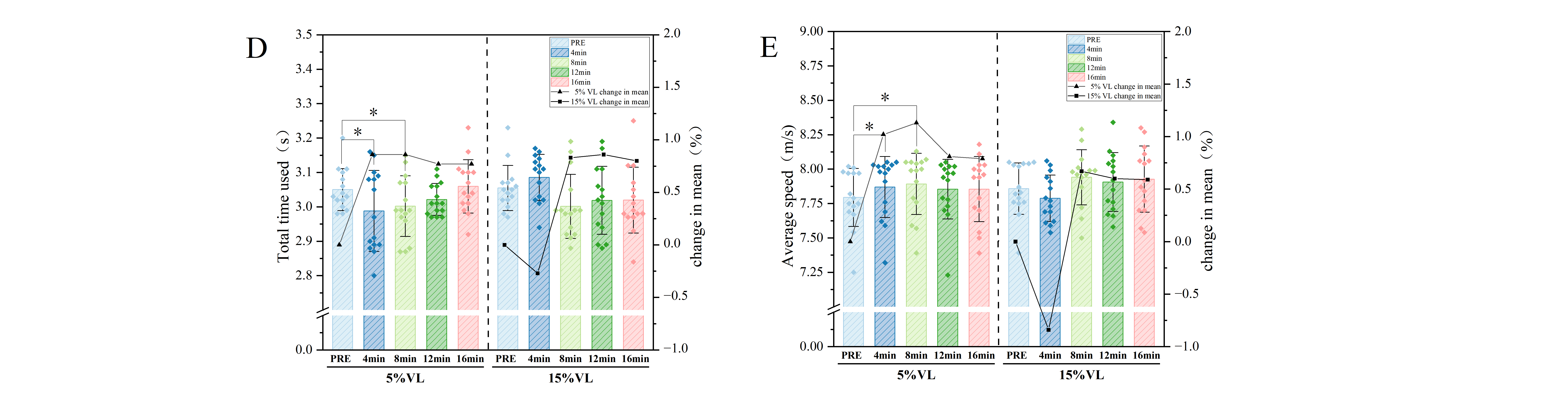


### Figure 3. Sprint performance following VL-controlled PAP protocols. (D: Total time used; E: Average speed) . Data are presented as mean ± SD. * indicates significant difference from PRE at p≤0.05.
